# Construction and screening of L-valine high-yielding *Escherichia coli* using an artificial screening marker

**DOI:** 10.3389/fmicb.2025.1627242

**Published:** 2025-08-07

**Authors:** Bowen Du, Sheng Gao, Daixue Kou, Yinuo Li, Dan Li, Yongsheng Cao, Cuiping Yang, Chuanzhuang Guo, Jianbin Wang, Junqing Wang, Nan Li

**Affiliations:** ^1^State Key Laboratory of Biobased Material and Green Papermaking (LBMP), Qilu University of Technology, Jinan, Shandong, China; ^2^School of Bioengineering, Qilu University of Technology, Jinan, Shandong, China; ^3^Dongxiao Bioengineering (Shandong) Co., Ltd., Zhucheng, China

**Keywords:** *Escherichia coli*, high-throughput screening, rare codon, L-valine, fluorescent protein

## Abstract

**Introduction:**

L-valine is commonly utilized in cosmetics, pharmaceuticals, food additives, and animal feeds. The selection and breeding of high-yielding, low-cost, and genetically stable production strains have become a key objective in the L-valine production industry.

**Methods:**

Using *Escherichia coli* DB-1-1, we developed a screening marker LESG associated with intracellular L-valine levels by choosing GTC, a less common codon for L-valine, in place of all L-valine codons. The artificial LESG was then ligated into pUC-57 and transformed into competent *E. coli* DB-1-1 cells with the rare L-valine codon. After conducting atmospheric and room-temperature plasma mutagenesis cultures, mutants that displayed elevated fluorescence were sorted using flow cytometry. After sorting the 240 strains. We sorted out 143 highly fluorescent strains, and the sorting efficiency reached 59.5%.

**Results:**

Fermentation results showed that the mutant strains with increased fluorescence intensity had an improved L-valine fermentation titer (23.1%) and a higher screening positivity rate (62.5%) than that of the wild-type strain. The maximum titer of valine at 24 h was 84.1 g/L.

**Conclusion:**

This approach offers a more comprehensive and effective method for identifying high-yielding L-valine bacterial strains.

## 1 Introduction

L-valine is one of the three branched-chain amino acids (leucine and isoleucine are the other two) needed to maintain animal health and metabolism; therefore, it is extensively added to food, medications, feed, and other items. It is mainly produced by microbial fermentation, the efficiency of which depends largely on the quality of the microorganisms (Li et al., 2017; Gao et al., 2021). L-valine promotes lactation when added to animal diets. For example, it is reported that adding L-valine to the diet of nursing sows and rats raises the levels of growth hormone and prolactin in their plasma (Roux et al., 2011; Corzo et al., 2007).

There are currently two main methods for L-valine production: chemical synthesis, and microbial fermentation. Direct extraction and chemical synthesis involve higher production costs and harsher reaction conditions than microbial fermentation (Ikeda, 2017). The low cost and use of renewable raw materials in the microbial production of L-valine has resulted in increased attention to improving the yield productivity of currently used processes, including developing high-yielding bacteria through mutation breeding methods, screening for nutritionally deficient strains, and using strains mutagenically treated with structural analogs of L-valine (Yang et al., 2023). However, structural analogs might cause complications with normal cell development as well as mutant survival. For example, screening of L-valine high-yielding strains after physical or chemical mutagenesis using the L-valine structural analog aminoethylcysteine has failed to effectively increase L-valine production (Ikeda, 2017). The application of traditional mutation breeding and nutrient-deficient strains for screening high-yielding strains is inefficient, and the utilization of metabolic engineering is unlikely to provide significant assistance in enhancing yields (Ma et al., 2020; Matsumoto et al., 2017; Bartek et al., 2011). Furthermore, these approaches are aimed at screening a specific gene, and the quantity of the screening mutation library is frequently low. If all the mutated genes are sorted out by this method, the workload will be extremely large, and correspondingly high-throughput screening methods have yet to be developed. As a consequence, a high-volumes approach for searching high-yielding amino acid isolates is required, one that combines accuracy and sensitivity while also being relevant to a diverse spectrum of bacteria. In recent years, the technique of atmospheric and room-temperature plasma (ARTP) has been frequently employed because it supports a high mutation rate, safety, and efficiency profile. Thus, ARTP could represent an effective genetic engineering tool for boosting L-valine production (Zhang et al., 2014).

Synthetic biology enables novel biosensing for high-titer strain selection by engineering rare codons into metabolic pathway genes. This leverages genetic code redundancy: synonymous codons exhibit different translation rates due to tRNA availability. Common codons, recognized by abundant tRNAs, enable efficient translation. Conversely, replacing common codons with rare codons can limit translation, especially when the cell is deficient in the corresponding amino acid. In deficiency, aminoacyl-tRNA synthetases struggle to charge rare tRNA isoacceptors, stalling translation. Genes with more rare codons are harder to express. When amino acids are sufficient and rare tRNAs are charged, translation resumes. Therefore, in industrial strains using this strategy, sufficient intracellular amino acid levels (indicating high production) allow normal translation despite codon bias. Strains achieving this are expected to be high-yield producers (Yu et al., 2015; Zheng et al., 2018).

To efficiently obtain strains with high L-valine production, we developed a new screening strategy, screening amino acid-producing strains by using rare codons. We linked fluorescent proteins to the target genes, used the rare codon GTC to replace the codon for L-valine in the gene fragments, and simulated the gene expression under different L-valine yields, so as to determine the link between L-valine levels and fluorescence intensity in the strains (Mohsin et al., 2015). ARTP mutagenesis and screening via high-throughput fluorescence-activated cell sorting (FACS) were used to identify strains with significant L-valine production. A high-yielding candidate strain was subsequently fermented in a 5 L fermenter and achieved an L- valine titer of 84.1 g/L in 24 h. Thus, we used a combination of artificially created rare codons combined with ARTP mutagenesis and high-throughput screening to provide an effective, accurate, and simple screening strategy for various microbial cultures.

## 2 Materials and methods

### 2.1 Bacterial strain plasmids and culture media

All strains and plasmids used in this study are listed in Table 1. *Escherichia coli* DH5α and BL21 (DE3) cells were grown in Luria–Bertani (LB) medium containing 10 g/L peptone, 10 g/L NaCl, and 5 g/L yeast extract at 37°C and pH 7.2 (Mustafi et al., 2012). The seed medium contained 10 g/L polypeptone, 5 g/L yeast powder, 2.5 g/L NaCl, 1 g/L glucose, and 6.5 g/L ground beef (Fang et al., 2024). The fermentation medium contained 6 g/L glucose, 0.019 g/L FeSO_4_, 0.004 g/L MnSO_4_.H_2_O, 1.125 g/L KCl, 2.2 g/L yeast powder, 1.25 g/L phosphoric acid, 0.01 g/L vitamin B3, and 0.41 g/L MgSO_4_. The *E. coli* strains were usually grown in a culture medium supplemented with 25 μg/mL ampicillin for growth.

**TABLE 1:** Strains and plasmids used in this study.

Strain/plasmid	Genetic characteristics	Source
*Escherichia coli* DB-1-1	Wild-type, starting strain	This lab
*E. coli* DH5α	Δ(lacZYA -argF) U169, deoR, recA1, endA1, hsdR17, supE44, gyrA96, relA1	Vazyme
*E. coli* BL21 (DE3)	λ(DE3[lacI, lacUV5-T7 gene1 ind1 sam7 nin5]), Δ(ompT-nfrA)885	Vazyme
*E. coli*-BLESG	*E. coli*-BL21, contains the fluorescent protein StayGold and levE CDS	This work
*E. coli*-LESG	*E. coli*-DB1-1, contains the fluorescent protein StayGold and levE CDS	This work
*E. coli*-DK2	*E. coli*-DB1-1, ARTP mutagenesis	This work
pUC-57	Shuttle expression vector containing the IPTG inducible Ptrc promoter	
pUC-57- LESG	pUC-57 containing Staygold and levE CDS	This work

IPTG, isopropyl β-D-1-thiogalactopyranoside.

### 2.2 Codon usage frequency analysis

Analysis of *E. coli* amino acid codons involved searching the National Centre for Biotechnology Information (NCBI) database^1^ (Piwowarek et al., 2020).

### 2.3 Construction of a fluorescent protein expression vector

In order to better sort high-yield strains, we need to limit the normal expression of genes as much as possible, so we screened out the protein sequences with the highest proportion of valine in the target strains and restricted their expression by replacing all L-valine codons with the rare L-valine codons. From the *E. coli* DB-1-1 genome sequence and its codon frequency, the gene sequences for extracellular proteins with the highest proportion of L-valine codons were screened, and the gene sequence of the green fluorescent protein Staygold was obtained from the NCBI database.

To construct the fluorescent expression vector, the L-valine codons in the levE CDS and StayGold genes were all replaced by the GTC codon (the replacement frequency is 23%). The gene fragment was obtained via gene fragment synthesis (Kingsley Biotechnology, Nanjing, China) and seamless cloned into the EcoRI-and HindIII-digested pUC-57 plasmid sites.

Finally, colonies were initially identified through colony PCR amplification, followed by nucleotide sequence verification sequencing to validate fusion construct integrity. And the fluorescent expression vector was named pUC-57-LESG.

### 2.4 Fluorescence screening system based on artificial rare codon construction

The plasmid pUC-57-LESG was respectively, introduced into *E. coli* DB-1-1 and *E. coli* BL21, and positive recombinant colonies were selected and transferred to LB growth medium with 25 μg/mL ampicillin. The culture was then incubated at 37°C with shaking at 200 rpm. Expression of the target gene was induced based on the optimized conditions from previous experiments.

The recombinant strain was grown and injected into 50 mL of LB medium supplemented with 25 μg/mL ampicillin, beginning at an OD_600_ of 0.3. The cells were put in a temperature-controlled incubator at 37°C and shook at 200 rpm. After 12 h, 0.6 mM isopropyl β-D-1-thiogalactopyranoside (IPTG) was added to the medium, followed by induction of florescent protein production at 25°C and 200 rpm for 12 h. Subsequently, florescence was measured and analyzed using a Biotek enzyme marker (Biotek Instruments, Inc., Winooski, VA, United States).

The method for determining cell density: Place 1 ml of the bacterial liquid in a UV spectrophotometer and measure it at a wavelength of 600 nm.

### 2.5 ARTP mutagenesis and FACS

The ARTP mutagenesis system generates a larger library of mutations than conventional mutagenesis. Four recombinant strains with StayGold screening marker mutations were inoculated into LB medium for culture (37°C, 200 rpm). At the logarithmic metaphase (OD_600_ ≈ 0.8), a 0.6 mM IPTG inducer was added. Subsequently, the culture conditions were adjusted to 25°C and expression induced at low-temperature for 10 h at 200 rpm. The induced bacterial liquid was taken and adjusted to OD_600_ ≈ 0.6–0.8 and 10 μL was absorbed and evenly coated on sterilized metal slides and subjected to ARTP irradiation treatment involving five gradient time points (1, 3, 5, 7, and 9 min). The plasma treatment parameters were incident power of 120 W, helium flow rate of 10 SLM, and working air pressure of 120 kPa. The treated slides were transferred to 1 mL of LB medium containing antibiotics and vortex eluted for 60 s. Then, 100 μL of the elution solution was spread on the selection medium. After culturing at 37°C for 24 h, the colony-forming units were counted. By comparing the colony survival rates of different treatment durations, the critical irradiation time with a fatality rate > 90% was determined, and based on this, the dominant mutant strains were screened and obtained. Three biological replicates were performed during colony counting to ensure data reliability. Candidate mutant strains with a survival rate of < 10% were selected and inoculated into 50 mL LB medium (containing 100 μg/mL ampicillin). They were cultured at 37°C at 200 rpm until OD_600_ ≈ 0.2 was reached, and then 0.5 mM IPTG was added and the culture transferred to 25°C for continuous induction for 12 h. Bacteria were collected from 1 mL of the culture by centrifugation, washed with potassium phosphate buffer (pH 7.4), resuspended to OD_600_ ≈ 1.0, and sorted by flow cytometry based on GFP fluorescence intensity (FACSAria III, BD Biosciences) using a nozzle pore size of 70 μm, injection pressure of 60 psi, and excitation/emission wavelengths of 488/535 nm. Data acquisition was carried out using Beckman Summit 5.2 software. The sorting threshold was determined through pre-experiments. The cell population with the top 0.01% fluorescence intensity was set as the enrichment target. The target cells were collected and inoculated into a 96-well cell culture plate (containing fresh LB medium). Monoclonal amplification was carried out in shaking culture at 37°C (200 rpm). Three technical replicates were used for each processing group to ensure the repeatability of the sorting results (Liu et al., 2021; Chen et al., 2015).

### 2.6 Fermentation of mutant *E. coli* strains

The selected 240 mutant strains were inoculated onto 96-well plates and incubated for 24 h in seed medium. At the same time, *E. coli* and mutant strains were added to 96-well plates comprising seed media for flow cytometry. *E. coli* DB-1-1 and ARTP mutant seeds were inoculated at 5% in 96-deep-well plates with fermentation media and cultured for 48 h at 37°C and 200 rpm. The quantity of L-valine in the solution used for fermentation was determined at 24 h using a biosensor analyzer. In three consecutive trials, bacteria from 96-well plates having the greatest L-valine synthesis were injected into liquid LB growth medium, and left to grow continuously at 37°C and 200 rpm. After 16 h, the seed solution was inoculated into 100 mL of fermentation medium at 10% inoculum. After 24 h of fermentation, measurements of the production fluid were obtained at 2-h intervals, and the average OD_600_ values and L-valine concentrations were calculated from the three experiments.

### 2.7 Quantitative method for L-valine concentration

This study employed the 2,4-dinitrofluorobenzene (DNFB) pre-column derivatization method for amino acid quantification pre-treatment. The specific procedure was as follows: 100 μL of the supernatant to be tested was mixed with an equal volume of carbonate buffer (0.5 mol/L, pH 9.0) to establish an alkaline reaction system. Subsequently, 100 μL of DNFB derivatization reagent (1% in acetonitrile) was added to initiate the nucleophilic substitution reaction. The reaction mixture was placed in a thermostatic shaker (60°C ± 0.5°C, 600 rpm) for precise thermal derivatization for 60 min to achieve characteristic labeling of aromatic amino groups. After the reaction system cooled to room temperature, a 7-fold volume of phosphate dilution solution (0.005 mol/L, pH 7.0) was added to terminate the reaction. Phase separation of the derivative products from precipitates was achieved via high-speed centrifugation (12,000 × *g*, 5 min). The final supernatant was purified through a microporous filter membrane (0.22 μm), transferred to an HPLC-specific sample vial, and stored protected from light at 4°C throughout for subsequent HPLC analysis.

Quantitative analysis was performed using a Shimadzu LC-20A high-performance liquid chromatography (HPLC) system. The chromatographic separation conditions were as follows: A Shim-pack GIST C18 column (5 μm, 4.6 × 250 mm) was used. The solvent system consisted of buffer phase A (20 mM sodium acetate, pH 4.6) and organic phase B (HPLC-grade methanol), forming an isocratic elution system (50:50, v/v). Instrumental parameters were set as: column oven temperature maintained at 40°C ± 0.5°C, mobile phase flow rate at 1.0 mL/min, UV detector wavelength optimized to 360 nm (corresponding to the characteristic absorption peak of DNFB derivatives), injection volume of 10 μL, and a total run time of 15 min (including a 5 min column equilibration period).

### 2.8 Batch fermentation in a 5 L reactor

The high-yielding strain obtained after mutagenic sorting was subjected to plasmid knockout that generated strain *E. coli* DK2, which was inoculated into LB medium for strain activation, and then inoculated into seed medium to obtain the seed liquid. The activated seed solution was inoculated into a 5 L fermenter at 10% inoculum volume, containing 3 L of fermentation medium, and incubated at 37°C. The pH of the medium was maintained in a range of 7.0–7.2 by adding ammonia on a flow-through basis, and the relative dissolved oxygen content of the fermentation broth was maintained at > 30% by controlling the rotational speed of the stirrer and the amount of ventilation. The fermentation time was 24 h (Liu et al., 2017). The OD_600_ and residual sugar concentration of the fermentation broth were measured every 2 h, and the L-valine content was measured at the end of fermentation via high-performance liquid chromatography. When the glucose concentration in the fermentation broth was lower than 10 g/L during the replenishment batch fermentation, 700 g/L glucose solution was added to maintain the residual sugar concentration at 5–10 g/L until the end of fermentation.

## 3 Results

### 3.1 Amino acid codon analysis

**FIGURE 1:**
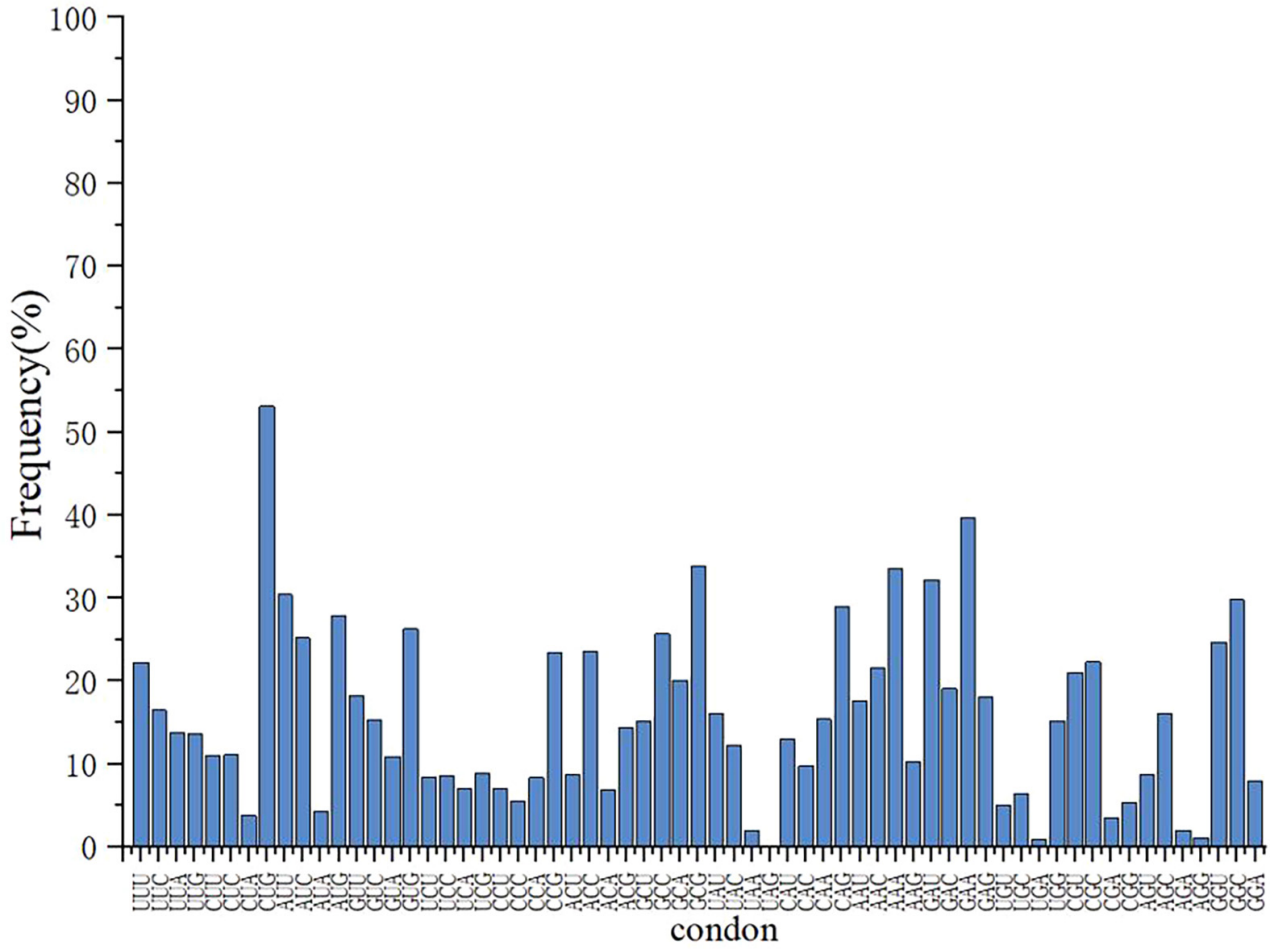
The frequency distribution of amino acid codon usage in the *E. coli* DB-1-1 genome.

**FIGURE 2:**
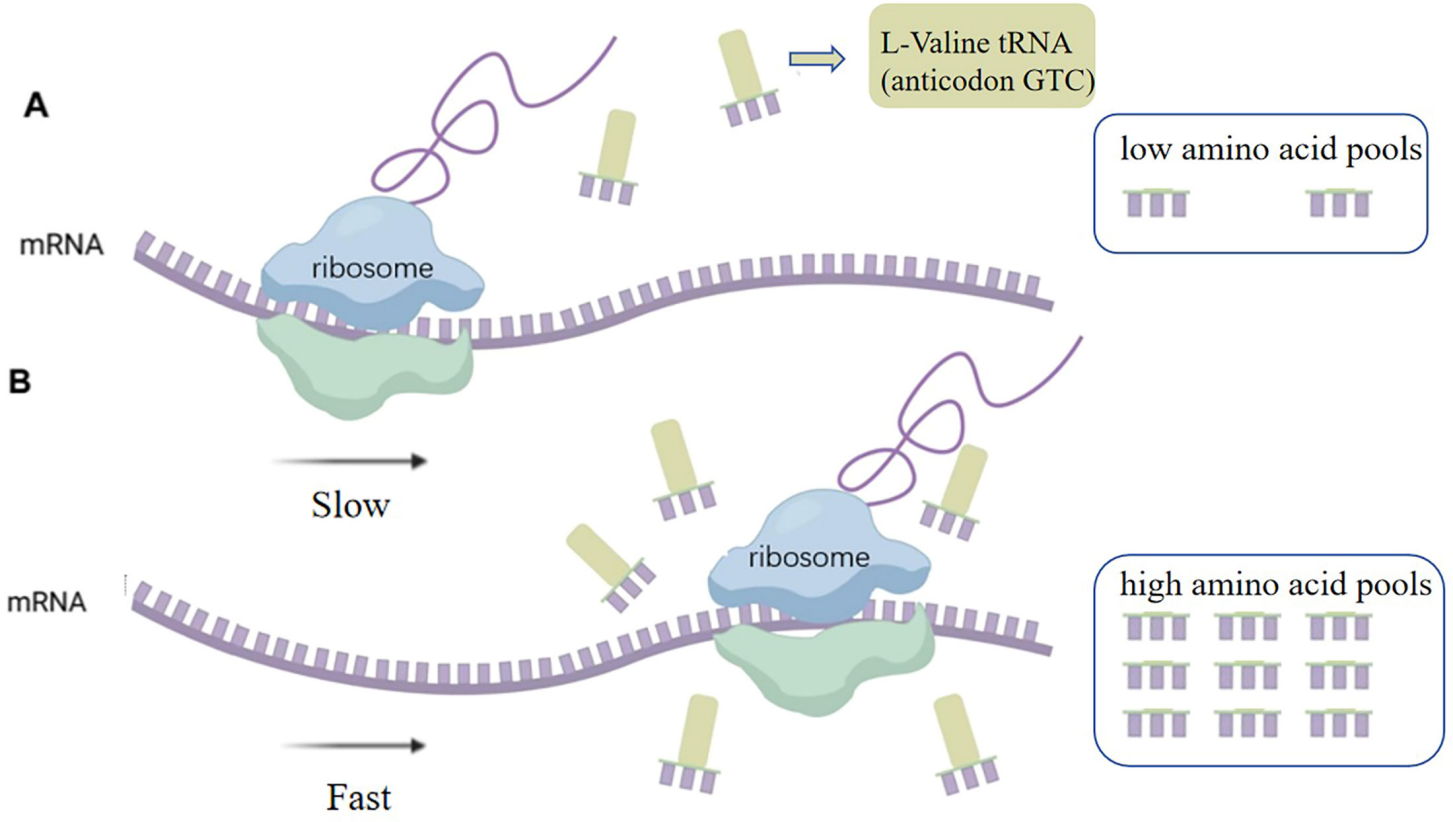
**(A)** Limited availability of rare-cognate tRNAs imposed translational bottlenecks, markedly attenuating heterologous protein synthesis efficiency. **(B)** Upon elevation of intracellular amino acid pools, aminoacyl-tRNA synthetases charge cognate tRNAs through stereospecific recognition, thereby reactivating translation elongation of transcripts containing programmed rare codons.

Analysis revealed that some codons were rare in *E. coli* DB-1-1 (Figure 1). For example, in the genome of *E. coli*, four codons encode threonine, of which ACA has a frequency of use of less than 6.9%, whereas four codons, GUU, GUC, GUA, and GUG, encode L-valine, with frequency distributions of 18.2%, 10.9%, 15.3%, and 26.3%, respectively. Therefore, the naturally rare L-valine codon in *E. coli* DB-1-1 is GUC. As a result, this codon may be utilized to screen for L-valine high-yielding strains using the rare-codon technique. The encoding of L-valine rare codons is regulated by the corresponding L-valine rare tRNAs and maintaining the related translation levels is especially important when L-valine is lacking in the cell. However, when the quantity of L-valine in the cell is high, the gene can be translated and expressed normally (Figure 2).

### 3.2 Expression of a fluorescent sorting system in *E. coli* BL21

**FIGURE 3:**
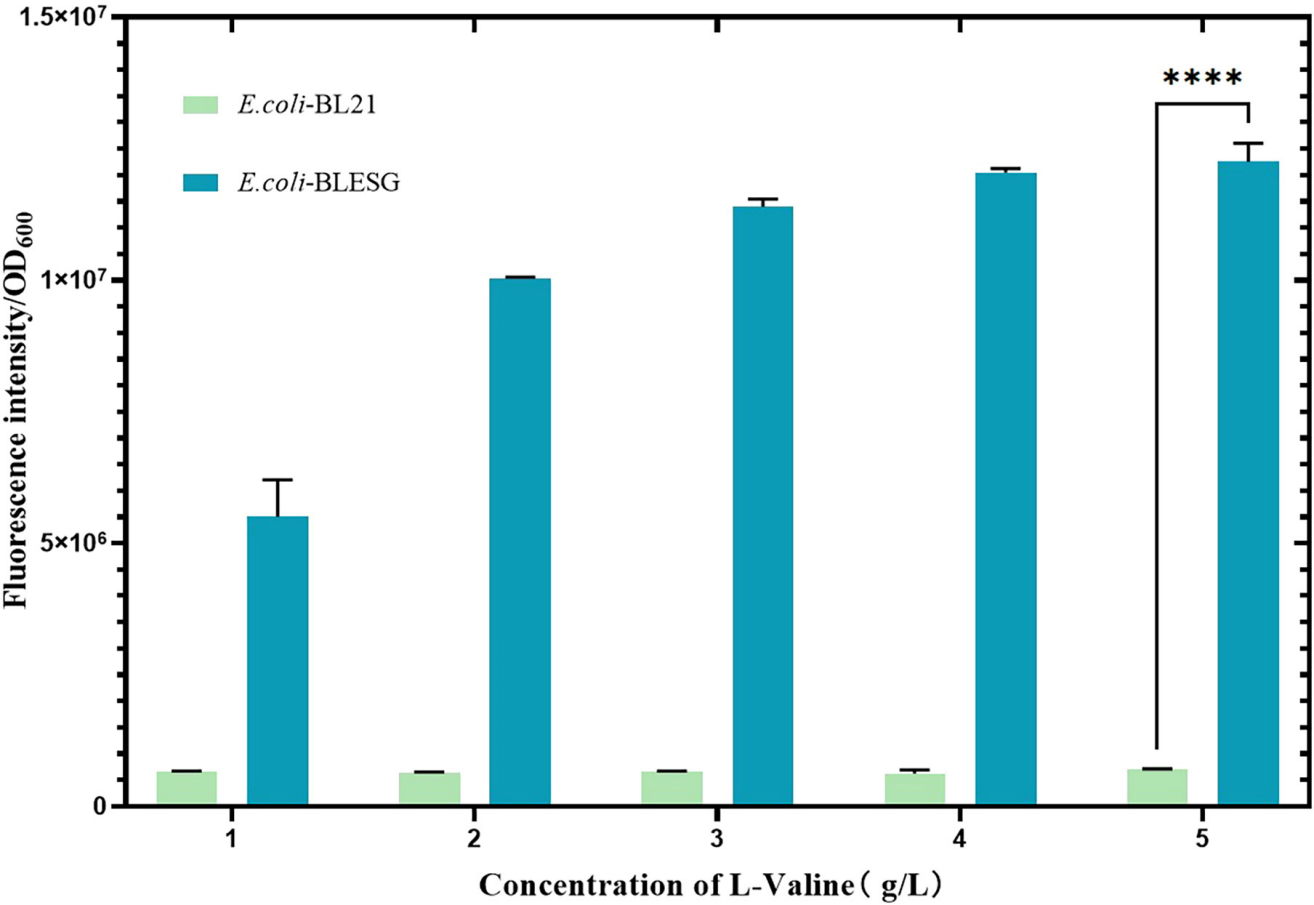
Fluorescence intensity of *E. coli* BLESG versus *E. coli* BL21 at varying L-valine supplementation concentrations. ****Represent significant landmark difference analysis.

The plasmid was transformed into pre-prepared *E. coli* BL21 host cells. Positive transformants, designated *E. coli*-LESG, were verified by PCR and electrophoresis (Supplementary Figure 1 and Supplementary Table 1). Following introduction of the fluorescent protein gene into *E. coli* BL21, expression was induced with IPTG. To confirm the positive correlation between the fluorescence intensity of the engineered strain and its L-valine production, we supplemented the medium with 1, 2, 3, and 4 g/L L-valine to simulate concentrations found in high-yielding strains. Results demonstrated that single-cell fluorescence intensity in *E. coli* LESG increased proportionally with L-valine concentration and was significantly higher than in wild-type *E. coli* BL21 (Figure 3). These findings confirm that plasmid pUC-57-LESG is functionally expressed in the strain, consistent with expected results.

### 3.3 Effect of transformation of fluorescent plasmids on *E. coli* DB-1-1

**FIGURE 4:**
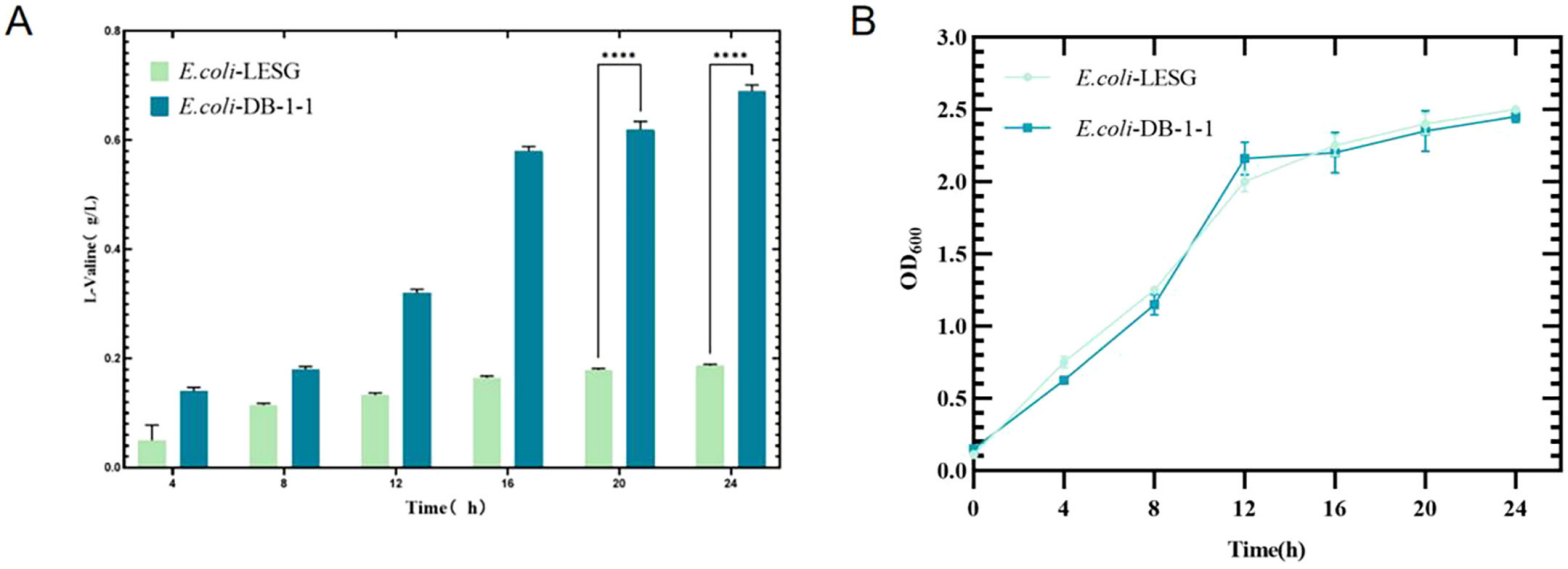
OD_600_ values and L-valine production in engineered strains of *E. coli*-LESG (fermentation medium). Samples were taken every 4 h from the fermentation medium of *E. coli*-LESG. **(A)** L-valine concentrations and **(B)** OD_600_ values were measured. Standard errors are shown as bars. ****Represent significant landmark difference analysis.

The plasmid was transfected into a pre-prepared *E. coli* DB-1-1 receptor cell. The positive strains, named *E. coli*-LESG, were verified using PCR and electrophoresis (Supplementary Figure 2 and Supplementary Table 2). The results showed that after the fluorescent protein particles were transfected into the recipient bacteria, the L-valine reference of the ferment solution was reduced by about 68.8% and the biomass was increased by approximately 4.1% compared with that of the wild-type *E. coli* DB-1-1 (*p* < 0.001) (Figure 4).

### 3.4 Expression of a fluorescent sorting system in *E. coli* DB-1-1

**FIGURE 5:**
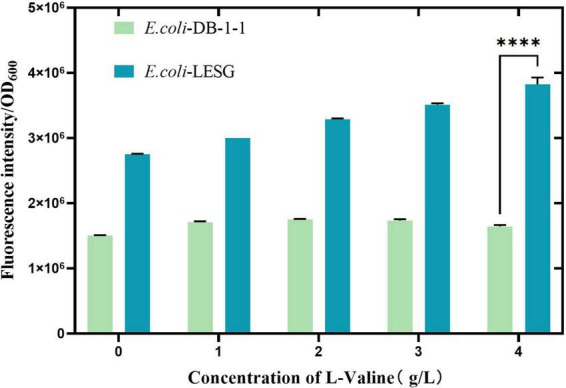
Fluorescence intensity of *E. coli*-LESG versus wild-type *E. coli* DB-1-1 at varying L-valine supplementation concentrations. ****Represent significant landmark difference analysis.

After the fluorescent protein gene was transferred into *E. coli* DB-1-1, its expression was induced using IPTG. The results showed that there was a significant increase in the fluorescence intensity of *E. coli*-LESG (*p* < 0.001), as compared with the wild type (Figure 5), suggesting that this gene is being expressed normally in *E. coli* DB-1-1. In order to verify the positive correlation between the fluorescence intensity of the target strain and its L-valine production, we added 1, 2, 3, and 4 g/L L-valine to the medium to simulate the concentration of L-valine in the high-yielding strains. The results showed that the fluorescence intensity of *E. coli*-LESG gradually increased with the addition of L-valine, whereas the fluorescence intensity of *E. coli* DB-1-1 was not changed appreciably. It can be concluded that the fluorescence intensity of *E. coli*-LESG was positively correlated with the production of L-valine.

### 3.5 Construction of mutant libraries using ARTP

**FIGURE 6:**
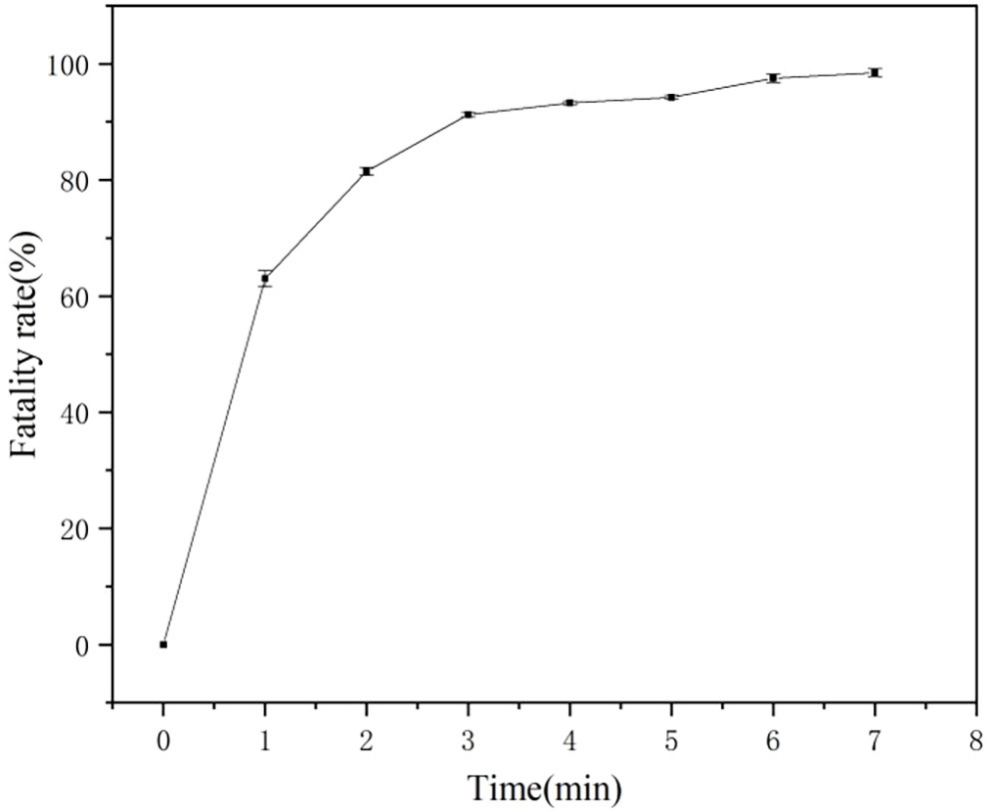
Atmospheric and room-temperature plasma (ARTP) mutagenesis fatality curve.

**FIGURE 7:**
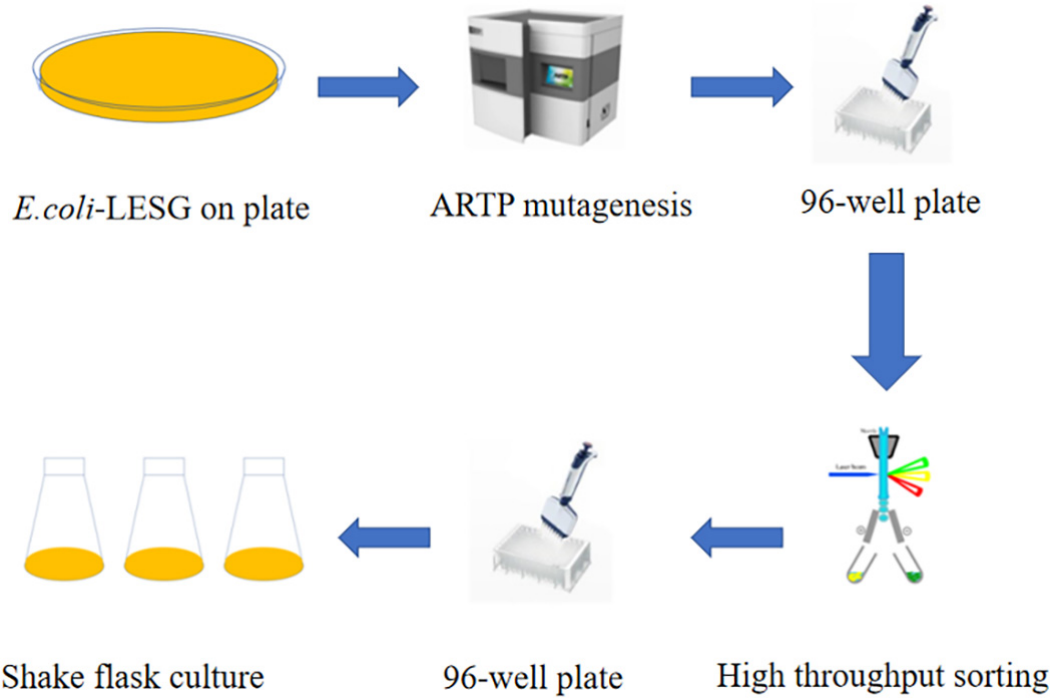
L-valine producing strains were obtained by ARTP mutagenesis and high throughput sorting.

We used the approach of Yang et al. (2023) of selecting of the best ARTP mutants with a mortality rate of more than 90% from preconditioning to sort and purify strains mutated by ARTP treatment for 5 min (Figure 6). Flow cytometry was used to perform ultra-high-speed sorting and purification of the mutant strains. The percentage of the total number of cells with high fluorescence to the total number of cells screened was approximately 0.01% (Supplementary Figure 3). Cells that showed the strongest fluorescence were subsequently sorted and inoculated into 96-well plates for the next step of culture and fermentation (Figure 7).

### 3.6 Fermentation of *E. coli* mutants

**FIGURE 8:**
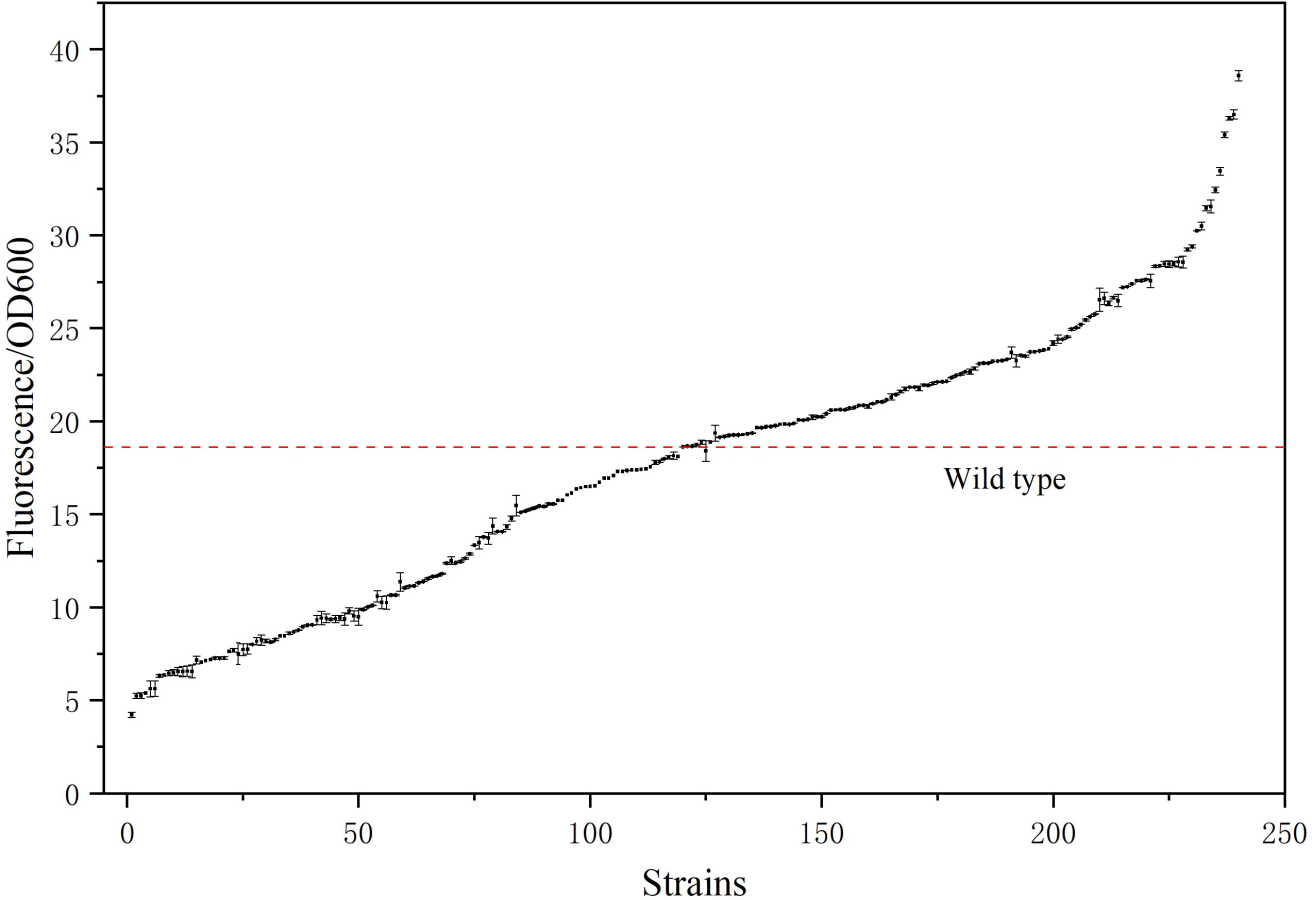
Fluorescence/OD_600_ ratio of the 240 strains sorted by flow cytometry. (The red line denotes the fluorescence intensity/OD_600_ ratio of the control strain).

**FIGURE 9:**
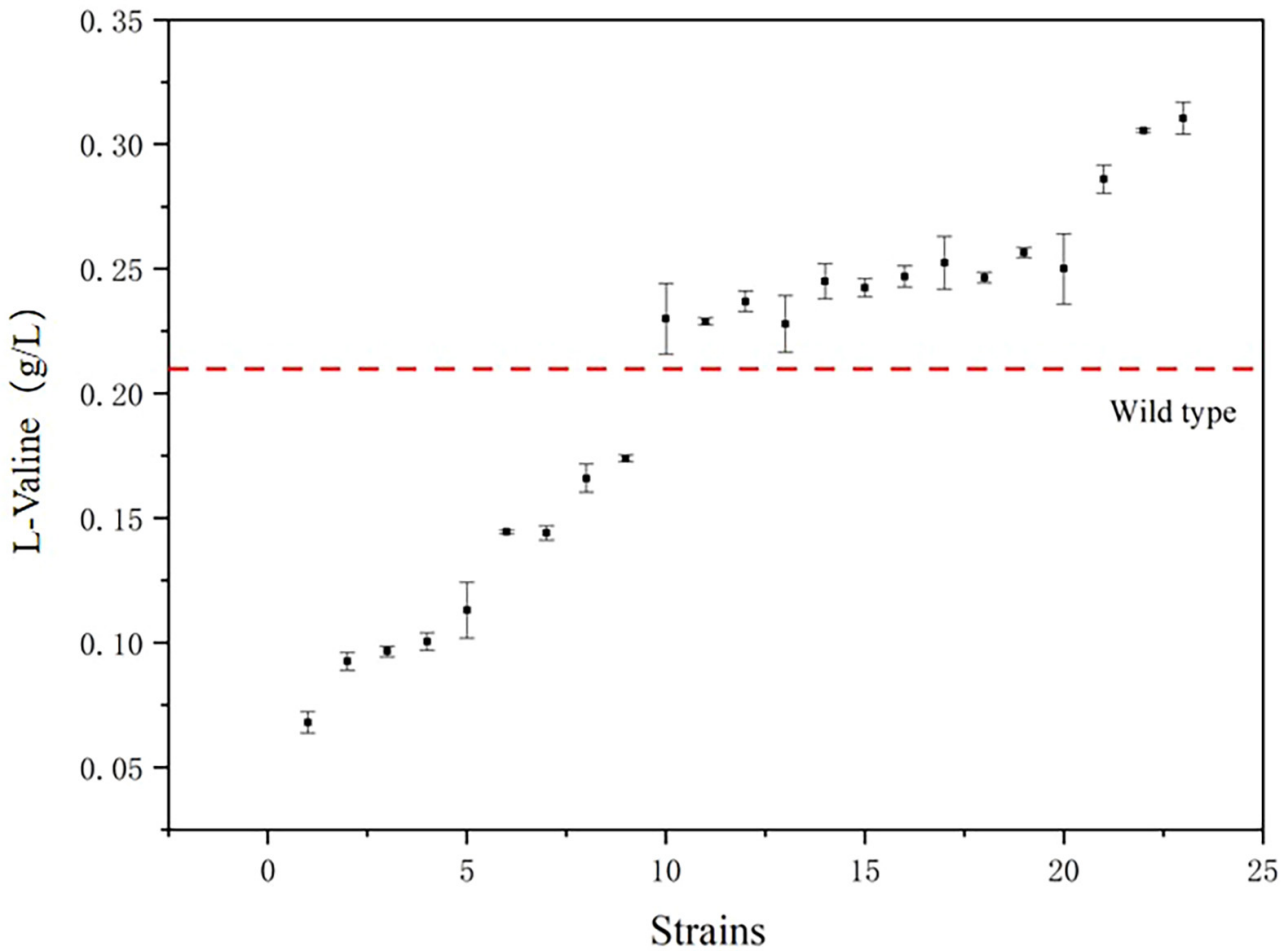
The titers of the 23 strains with the highest fluorescence intensity/OD_600_ value. (The red line segments represent the titers of the control strains).

**FIGURE 10:**
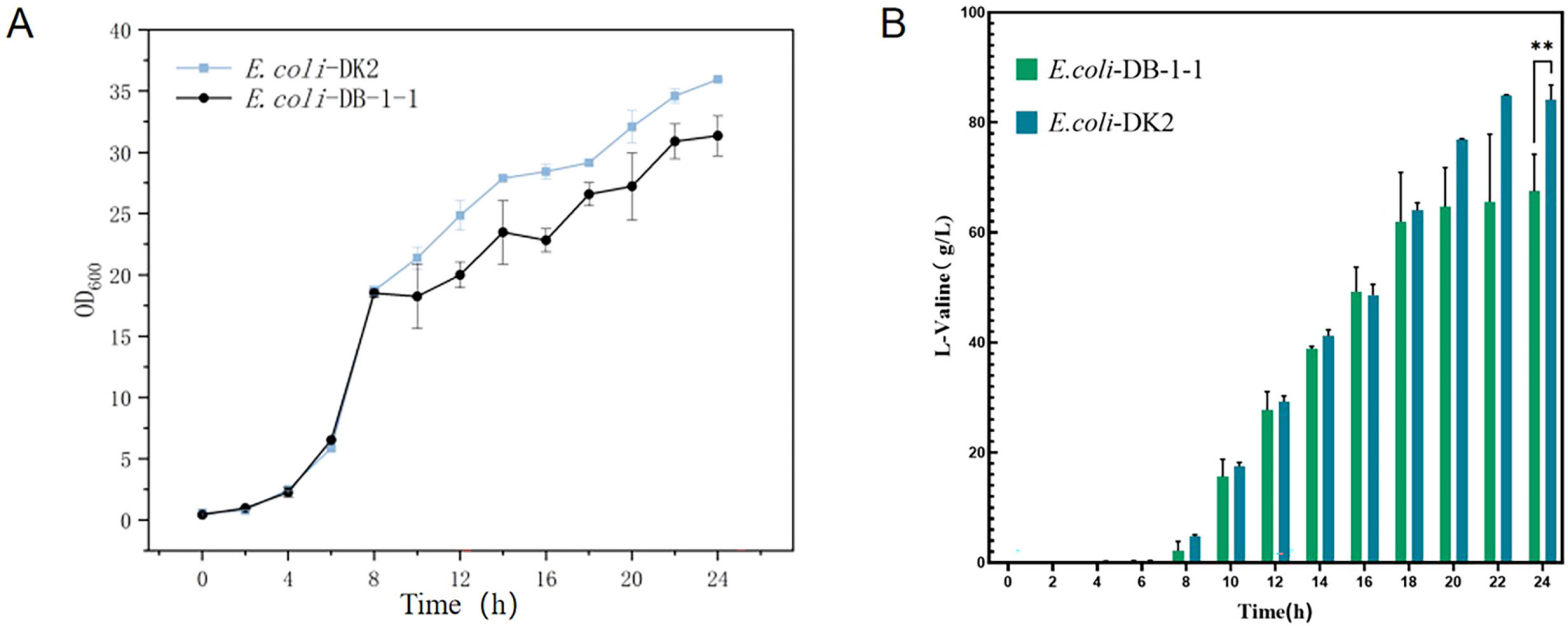
**(A)** OD_600_ values and **(B)** L-valine titer in the engineered strain of *E. coli*-DK2 (fermentation medium). **Represent significant landmark difference analysis.

The mutant bacteria were cultured in 96-well plates, and their fluorescence intensity and OD_600_ values were measured (Figure 8). In four 96-well plates cultured with *E. coli*-LESG mutants, 39, 40, 39, and 25 respective highly fluorescent strains were obtained. The screening efficiencies for each plate were 39/60 (65%), 40/60 (71%), 39/60 (65%), and 25/60 (42%), respectively, resulting in an average screening efficiency of about 59.5%. Among the rescreened high fluorescent *E. coli*-LESG mutants, 23 mutant strains were selected for fermentation culture (Figure 9). Fifteen strains were obtained from the rescreening (screening efficiency 65.2%), and the highest yielding strain was subjected to plasmid knockout treatment to obtain strain *E. coli*-DK2. Fermentation of this strain in a 5-liter fermenter resulted in a 23.1% increase in the titer of L-valine, up to 84.1 g/L as compared to the fermentation using *E. coli* DB-1-1 (*P* = 0.0027) (Figure 10).

## 4 Discussion

Currently, the common strains used for industrial production of L-valine are *E. coli* and *Corynebacterium glutamicum*. *Escherichia coli* is the most thoroughly studied strain and one of the most popular strains for the industrial production of L-valine, with the advantages of rapid multiplication and easy control of metabolism (Wang et al., 2018; Oldiges et al., 2014). However, there are fewer reports of L-valine production by *E. coli* compared to *C. glutamicum* strains. Microbial fermentation is one of the major modes of L-valine production and, to increase the yield, mutagenesis and metabolic engineering strategies have been used (Schwentner et al., 2018; Hasegawa et al., 2012). Chen et al. (2015) metabolically engineered *Corynebacterium glutamicum* to produce 437 mM (51.2 g/L) L-valine after 96 h of fermentation. Hao et al. (2022) modified *Escherichia coli* through metabolic engineeringIn the 5-L bioreactor, the L-valine production of the strain was 92 g/L. Despite advances in production strain improvement, there is still no effective means to screen for high-titer L-valine-producing strains.

Rare codons are increasingly used in strain screening as an alternative to codon optimization. By replacing common codons with rare counterparts in heterologous genes, translation can be suppressed–especially under amino acid deficiency–due to inefficient charging of rare tRNAs by aminoacyl-tRNA synthetases (aaRS), stalling ribosome elongation (Guimaraes et al., 2020). When intracellular amino acid levels rise sufficiently for aaRS to charge rare tRNAs, translation resumes. Industrial strains engineered with rare codons thus exhibit normal translation only when amino acid production is high, directly linking rare-codon translation efficiency to yield (Zheng et al., 2018).

This principle extends to artificial codon systems: Zheng et al. (2018) observed *E. coli* growth impairment when artificial codons lacked cognate tRNAs, demonstrating programmable control over translation. Huo et al. (2019) leveraged this by increasing rare-codon density in sensor genes, raising the amino acid concentration threshold required for translation to establish a high-throughput screen for amino acid-overproducing strains in *E. coli* and *C. glutamicum*.

Here, we describe a method for screening strains for high L-valine production by constructing a rare codon system. Yang et al. (2023) artificially constructed the L-lysine rare codon AAA by replacing the lysine tRNA promoter with the non-inducible promoter Parg. Unlike the case for lysine, a naturally occurring rare codon GTC exists in L-valine. We exploited this codon to demonstrate a relationship between intracellular L-valine levels and fluorescence intensity. To create high-yielding L-valine strains, ARTP mutagenesis was combined with high-throughput fluorescence-activated cell sorting.

Currently, L-valine titer in *E. coli* reaches 92 g/L in 48 h, which is the best available standard, achieved after ARTP mutagenesis combined with high-throughput screening for enhanced L-valine carbon flux and output, optimized expression of transcription factors, and intracellular cofactor homeostasis (Hao et al., 2022). However, the highest titer in 24 h to date was only 32.6 g/L. The productivity at 48 h was only 1.916 g/L/h. In the present study, the highest titer of L-valine in 24 h was 84.1 g/L, the productivity has even reached 3.5 g/L/h, which is a significant improvement. This study provides an effective method for screening microbial strains that can produce high amounts of multiple amino acids. The final high-titer strain *E. coli*-DK2 obtained in this study showed a 23.1% increase in L-valine yield. However, there are remaining issues that require further research. For example, only one mutagenesis method was used in this study, so the mutation library can be expanded through multiple rounds of mutagenesis or a combination of physical and chemical mutagenesis. In addition, a promoter-optimization strategy may be employed to enhance the synthetic pathway by optimizing the promoters of key enzymes in the L-valine synthesis process and further optimizing the fermentation conditions in the fermenter as a means to increase the yield of L-valine.

## 5 Conclusion

In conclusion, we have developed a comprehensive and effective method for identifying high-yielding L-valine bacterial strains by using rare codons to replace L-valine codons in target high-L-valine-producing genes, so as to limiting heterologous protein expression and selectively express these genes, and subsequently detect them by fluorescence. The approach is widely applicable as it can be adapted for screening microbial strains with a high production of multiple amino acids. And this approach can be combined with methods such as metabolic engineering and mutation breeding.

## Data Availability

Publicly available datasets were analyzed in this study. This data can be found here: https://www.ncbi.nlm.nih.gov/datasets/genome/GCF_013167015.1/.

## Footnotes

1
https://www.ncbi.nlm.nih.gov/
