# Supplementary material for: Construction and screening of L-valine high-yielding Escherichia coli using an artificial screening marker
Source: Front Microbiol. 2025 Aug 7;16:1627242. doi: 10.3389/fmicb.2025.1627242 (PMC12369413; doi:10.3389/fmicb.2025.1627242)
Supplement: Supplementary file 1 [file Supplementary_file_1.docx]

**Supplementary Table 1. Primers used in this study.**

| Primer | Sequence (5′→3′) | Restriction site |
| --- | --- | --- |
| P1 | AAATCGAAACCCAGCCGGCT | *Hin*dIII |
| P2 | AAAATGTAGTTCGTGCTACGCACACCAT |  |
| P3 | AAGCACAACCGCGTGCAGAGT |  |
| P4 | ATTTGCTAAAGCGGCGCCAATT | *Eco*RI |

**Supplementary Table 2. Primers used in this study.**

| Primer | Sequence (5′→3′) | Restriction site |
| --- | --- | --- |
| P1 | CGCCCTTATTCCCTTTTTTGCGG | *Hin*dIII |
| P2 | TCACCGGCTCCAGATTTATCAGC |  |
| P3 | CCCTTTTTTGCGGCATTTTGCCTTC |  |
| P4 | TTGCCTGACTCCCCGTCGTGTA | *Eco*RI |

**Supplementary Fig 1.**


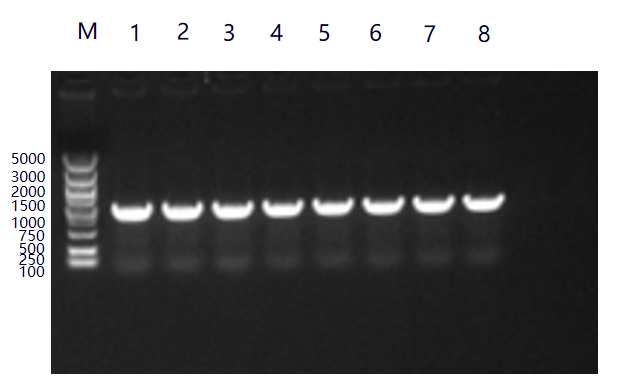


**Supplementary Fig 1.Agarose gel electrophoresis results of Staygold-levE fragments**

**Supplementary Fig 2.**


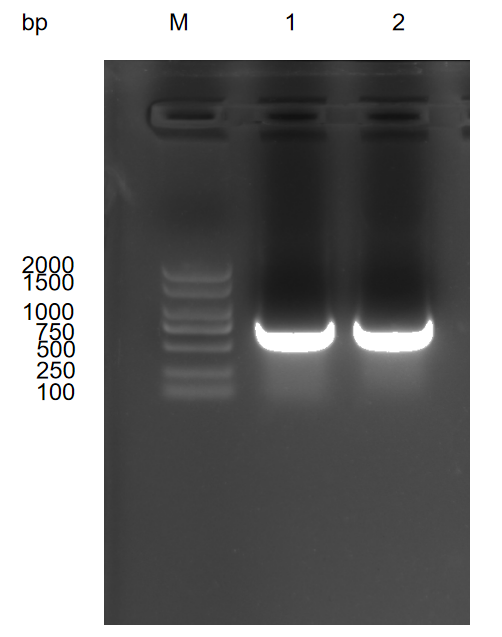


**Supplementary Fig 2.Agarose gel electrophoresis results of Staygold-levE fragments**


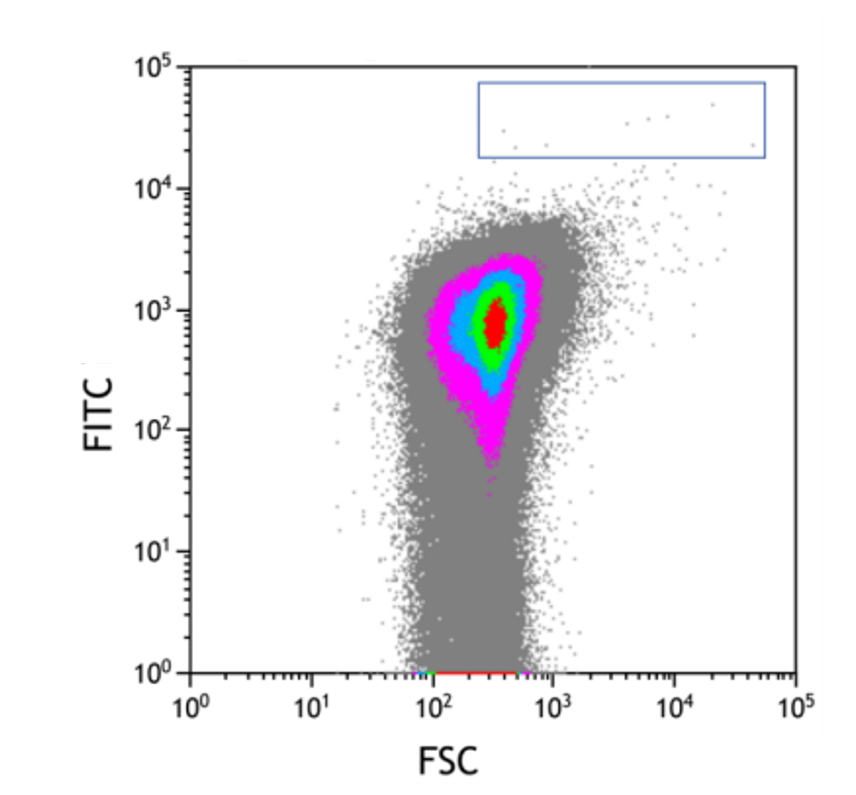


**Supplementary Fig 3.Flow cytometry analysis results.**
